# Cloning and Transcriptional Activity of the Mouse *Omi*/*HtrA2* Gene Promoter

**DOI:** 10.3390/ijms17010119

**Published:** 2016-01-16

**Authors:** Dan Liu, Xin Liu, Ye Wu, Wen Wang, Xinliang Ma, Huirong Liu

**Affiliations:** 1 Department of Physiology and Pathophysiology, School of Basic Medical Sciences, Capital Medical University, Beijing 100069, China; liudanzyh@126.com (D.L.); liuxintea@126.com (X.L.); wuyecat530@126.com (Y.W.); wangwen@ccmu.edu.cn (W.W.); 2 Beijing Key Laboratory of Metabolic Disturbance Related Cardiovascular Disease, Beijing 100069, China; 3 Department of Emergency Medicine, Thomas Jefferson University, Philadelphia, PA 19107, USA

**Keywords:** Omi/HtrA2, promoter, transcriptional activity, luciferase

## Abstract

HtrA serine peptidase 2 (HtrA2), also named Omi, is a pro-apoptotic protein that exhibits dramatic changes in expression levels in a variety of disorders, including ischemia/reperfusion injury, cancer, and neurodegeneration. In our study, Omi/HtrA2 protein levels were high in the heart, brain, kidney and liver, with elevated heart/brain expression in aging mice. A similar expression pattern was observed at the mRNA level, which suggests that the regulation of Omi/HtrA2 is predominately transcriptional. Promoter binding by transcription factors is the main influencing factor of transcription, and to identify specific promoter elements that contribute to the differential expression of mouse Omi/HtrA2, we constructed truncated Omi/HtrA2 promoter/luciferase reporter vectors and analyzed their relative luciferase activity; it was greatest in the promoter regions at −1205~−838 bp and −146~+93 bp, with the −838~−649 bp region exhibiting negative regulatory activity. Bioinformatics analysis suggested that the *Omi/HtrA2* gene promoter contains a CpG island at −709~+37 bp, and eight heat shock transcription factor 1 (HSF1) sites, two Sp1 transcription factor (SP1)sites, one activator protein (AP) site, seven p53 sites, and four YY1 transcription factor(YY1) sites were predicted in the core areas. Furthermore, we found that p53 and HSF1 specifically binds to the Omi/HtrA2 promoter using chromatin immunoprecipitation analysis. These results provide a foundation for understanding Omi/HtrA2 regulatory mechanisms, which could further understanding of HtrA-associated diseases.

## 1. Introduction

Omi/HtrA2, a mitochondrial pro-apoptotic protein, is member of the High Temperature Requirement (HtrA) family. Abnormal gene expression and localization of Omi/HtrA2 has been associated with a variety of disease. For instance, Omi/HtrA2 expression is elevated in lung adenocarcinoma [1], hepatocellular carcinoma [2,3], and squamous cell carcinoma of the head and neck [4]. On the other hand, reduced expression has been observed in small lymphocytic lymphoma/chronic lymphocytic leukemia (SLL/CLL) [5], testicular germ cell cancer [6] and ovarian cancer [7,8,9,10]. Furthermore, overexpression of mature Omi/HtrA2, results in increased matrix metallopeptidase 3 (MMP-3) activity and cell death in dopaminergic cells [11]. We previously reported that overexpression of Omi/HtrA2 also contributes to apoptosis, and increases the susceptibility of myocardial ischemia reperfusion injury in aging rat hearts [12,13]. All the above reports are consistent with the possibility that alterations in Omi/HtrA2 transcription and protein levels are closely associated with disease. Consequently, it is important to study the mechanisms of transcriptional regulation of Omi/HtrA2.

Promoters, which are located upstream of genes, are key points for modulating transcriptional levels. Gene promoters have many transcriptional binding sites, which can bind specific transcription factors and influence the mRNA expression levels and chromatin organization of the gene. CpG islands are DNA sequences that are rich in CG nucleotides. They often are observed around the promoter region and the first exon of genes, where they are associated with major epigenetic changes such as methylation, which influences the expression of the associated genes. Therefore, it is important to consider both the transcription factor binding sites and the potential occurrence of CpG islands to obtain a multilevel understanding of the regulatory mechanism of genes.

In our study, we amplified the promoter of the mouse *Omi*/*HtrA2* gene and analyzed its sequence using bioinformatics (Network Promoter Prediction, CpG Island Searcher, TFSEARCH, JAPAR, PROMO). To study the core region of the promoter, we constructed an Omi/HtrA2 promoter/luciferase reporter vector, as well as several deletion mutants, which we transfected into murine fibroblast cells (NIH3T3), rat myocardial cells (H9c2) and human embryonic kidney cell (HEK-293) cells. This research provides a theoretical basis for study of the transcriptional regulation and function of the *Omi*/*HtrA2* gene.

## 2. Results

### 2.1. HtrA Serine Peptidase 2 (Omi/HtrA2) is Ubiquitously Expressed but Its Protein and mRNA Levels Vary in Different Tissues

**Figure 1:**
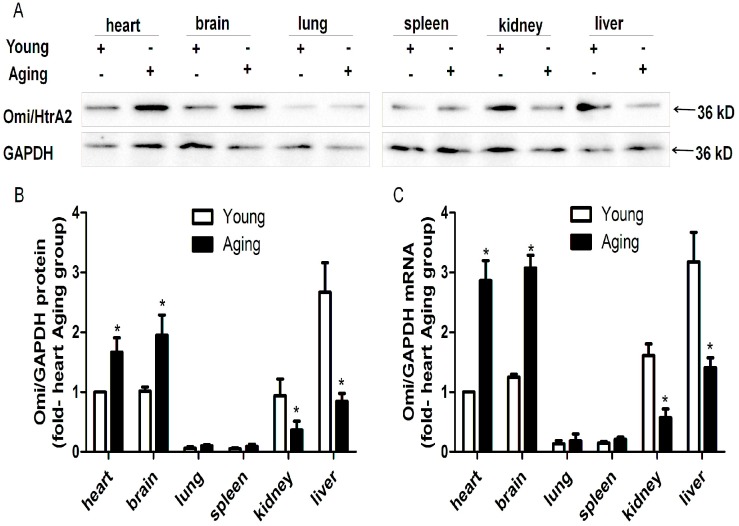
Tissue specific expression of HtrA serine peptidase 2 (Omi/HtrA2). Omi/HtrA2 protein expression in young and aging mice was detected by Western blotting (**A**), and the relative levels were quantified using ImageJ software (**B**). Omi/HtrA2 mRNA expression was detected by quantitative RT-PCR (**C**). *n* = 5 per group. * *p* < 0.05 *vs.* young.

To determine whether Omi/HtrA2 is differentially expressed in tissues of young and aging mice, we performed Western blotting. For both the young and aging mice, Omi/HtrA2 showed a pattern of high protein expression in the heart, brain, kidney and liver, and lower expression in the lung and spleen. However, the balance of the expression appeared to shift, with the greatest expression in the kidney and liver for the young mice and the greatest expression in the heart and brain for the aging mice (Figure 1A,B). The variable expression of Omi/HtrA2 indicates that its regulation is complex, involving both tissue-specific and age-specific factors.

To determine whether the differential expression of mouse Omi/HtrA2 is regulated at the level of transcription, we assessed mRNA levels by QRT-PCR (Figure 1C). A similar pattern of high expression in the kidney and liver for young mice and the heart and brain for aging mice was observed. These results verify the Western blotting results and suggest that the regulation of differential Omi/HtrA2 expression is likely to occur at the level of transcription and to involve a complex dynamic of regulatory factors.

### 2.2. Assessment of the Activity of Mouse Omi/HtrA2 Promoter Luciferase Full-Length and Truncated Vectors 

**Figure 2:**
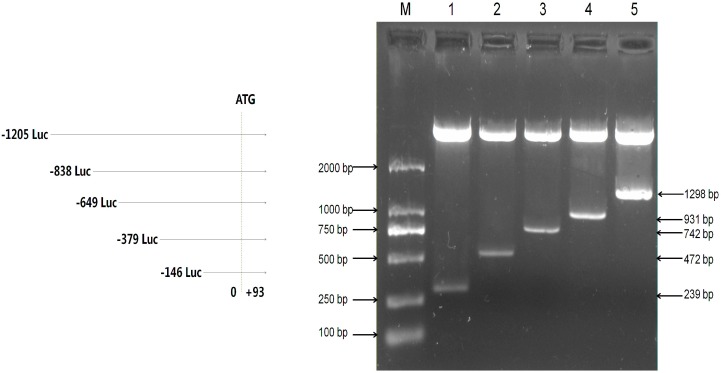
Verification of mouse Omi/HtrA2 promoter luciferase expression plasmids. Plasmids were verified by double enzyme digestion with *Kpn*I and *Hind*III, which releases a vector fragment and a variable-sized promoter insert. M: DL2000 Marker; **1**: pGL3-239; **2**: pGL3-472; **3**: pGL3-742; **4**: pGL3-931; **5**: pGL3-1298. Luc: luciferase expression plasmids.

To analyze transcriptional activity of the mouse *Omi*/*HtrA2* gene promoter, we amplified different lengths of the mouse *Omi*/*HtrA2* gene promoter by PCR. Five products were amplified using F1–F5 as upstream primers with R as a downstream primer (Table 1). The predicted products were verified by 1% agarose gel electrophoresis (Figure S1) followed by sequencing. The five truncated PCR products were cloned into PMD-18T, and then subcloned into the pGL3 luciferase reporter vectors (pGL3). The resulting five luciferase reporter plasmids (named pGL3-239, pGL3-472, pGL3-742, pGL3-931, and pGL3-1298 according to length) were confirmed by *Kpn*I and *Hind*III digest (Figure 2).

**Table 1:** Primer sequences of different lengths of the mouse *Omi*/*HtrA2* gene promoter. R is the shared reverse primer, and F1, F2, F3, F4 and F5 represent the forward primers for PCR fragments with different length.

Primer Name	Sequence (5’–3’)	Amplification Region (bp)	Length (bp)
F1	AGAATTTCGAGGGCAGGGCTAA	−146~+93	239
F2	CGTGAGGTGGCGATTAAG	−379~+93	472
F3	TGGAAATAAGGACCCTGACG	−649~+93	742
F4	GTAGAGCAGTAGCGCGAGCA	−838~+93	931
F5	CTGGGAAGGCGGAGTCTTT	−1205~+93	1298
R	GGCAAGCGGTCTAGGAGAA	–	–

**Figure 3:**
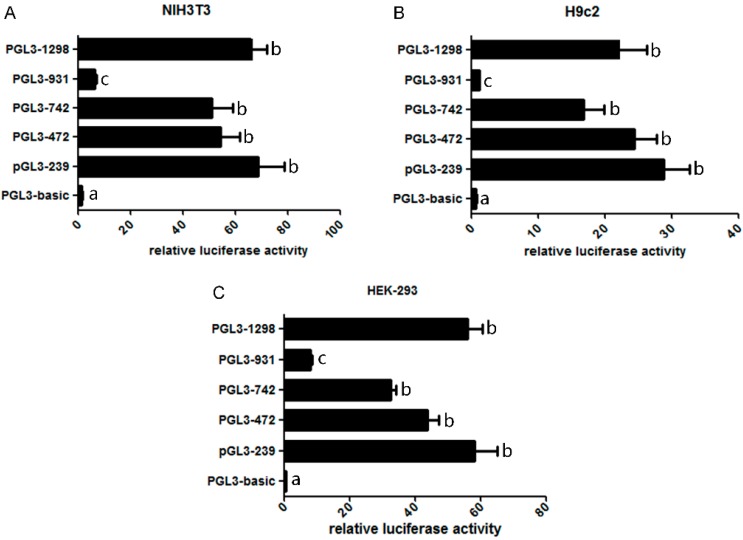
Relative luciferase activity of full length and truncated mouse Omi/HtrA2 promoter plasmids. Luciferase assays were performed in NIH3T3 cells (**A**); H9c2 cells (**B**); and HEK-293 cells (**C**). The different letters indicate the significant differences between the plasmids (*p* < 0.05).

To identify the core regions of the mouse *Omi*/*HtrA2* gene promoter, we transfected the luciferase vector pGL3-basic (no insert) and the five truncated *Omi*/*HtrA2* gene promoter luciferase plasmids into NIH3T3, H9c2 and HEK-293 cells, with pRL renilla luciferase control reporter vector (pRL-TK) as internal control. Relative luciferase activity (RLA) as an index of promoter activity was tested 48h after transfection using a dual-luciferase reporter assay system (Figure 3). Significant differences between the two transfection groups suggested that the core activity resides within the truncated sequence; results from the three cell lines were similar. Most of the luciferase reporter vectors, including pGL3-239 (−146~+93 bp), pGL3-472 (−379~+93 bp), pGL3-742 (−649~+93 bp), pGL3-931 (−838~+93 bp) and pGL3-1298 (−1205~+93 bp), had greater activity than pGL3-basic. The RLA of pGL3-1298 (−1205~+93 bp) and pGL3-239 (−146~+93 bp) was significantly higher than pGL3-931 (−838~+93 bp) and pGL3-basic, suggesting that the core activity resides within the −1205~−838 bp and −146~+93 bp regions (truncated sequence) of the *Omi*/*HtrA2* gene promoter. The activities of pGL3-472 (−379~+93 bp) and pGL3-742 (−649~+93 bp) were not significantly different from that of pGL3-239 (−146~+93 bp). Furthermore, the activity of pGL3-931 (−838~+93 bp) was lower than pGL3-742 (−649~+93 bp) suggesting that a negative regulatory element exists in the −838~−649 bp region of the Omi/HtrA2 promoter.

### 2.3. Bioinformatics Analysis of the Mouse Omi/HtrA2 Promoter

In order to investigate the transcriptional factors that regulate the expression of mouse Omi/HtrA2, bioinformatics analysis was performed using online tools.

Network Promoter Prediction assessed the core areas of the Omi/HtrA2 promoter, and two potential core active regions (−359~−310 bp and −1197~−1148 bp) with relatively high scores were identified in this promoter sequence. One CpG island was predicted in this promoter, which was located from −709 to +37 bp (Table 2).

Several potential transcription factor binding sites were predicted in the promoter of the *Omi*/*HtrA2* gene by using online tools TFSEARCH, JAPAR and PROMO. This includes eight Heat shock transcription factor 1 (HSF1) sites, two SP1 sites, one AP site, seven p53 sites, four YY1 sites (Table 3).

**Table 2:** Prediction of core area and CpG Island of Mouse Omi/HtrA2 promoter.

Prediction Area	Start	End	Promoter Sequence	Score
promoter core area 1	−1197	−1148	GAGGGCAGGGCTAAAAGTGGGCAGACAGGAAAGGAACTAGGGCACCCACT	0.98
promoter core area 2	−359	−310	CCGGTGCGAGTCAAAGAGCCGCTCCGGCCCCGGAGCTGGGGGAGGTTCCA	0.90
CpG island	−709	+37	Observed/Expected ratio > 0.60	Percent C + Percent G > 50.00

**Table 3:** Analysis of transcription factor binding sites of the Omi/HtrA2 promoter.

Transcription Factors	Number	Sequence of Putative Binding Sites	Location
HSF1	1	AAGGAACT	+83~+90
	2	GTGGAAGC	−46~−39
	3	CTTCTTGCTTTTTCT	−172~−158
	4	ATGGAAGG	−181~−174
	5	GGGGAACA	−285~−278
	6	AGGGAAGA	−805~−798
	7	ATGGAACC	−868~−861
	8	CACAGAAT	−1028~−1021
SP1	1	GCCTCGCCCC	−722~−713
	2	CACCCGCCTAT	−999~−899
AP1	1	CTATTGA	−944~−938
p53	1	CGTGCCC	+65~+71
	2	GCGGCCC	−54~−48
	3	GGGCTAG	−84~−78
	4	GGGCATC	−235~−229
	5	CCAGCCCAGCCC	−520~−509
	6	GGGCTGG	−816~−810
	7	GCAGCCC	−1103~−1197
YY1	1	GAAAAGACA	−867~−859
	2	AGGGTGACA	−843~−835
	3	CTGGGGACA	−551~−543
	4	TGTCGCCGC	−177~−169

HSF1: Heat shock transcription factor 1; AP1: activator protein 1; SP1: Sp1 transcription factor; YY1: YY1 transcription factor.

### 2.4. p53 and HSF1 Directly Binds to Omi/HtrA2 Promoter with ChIP Assay

**Figure 4:**
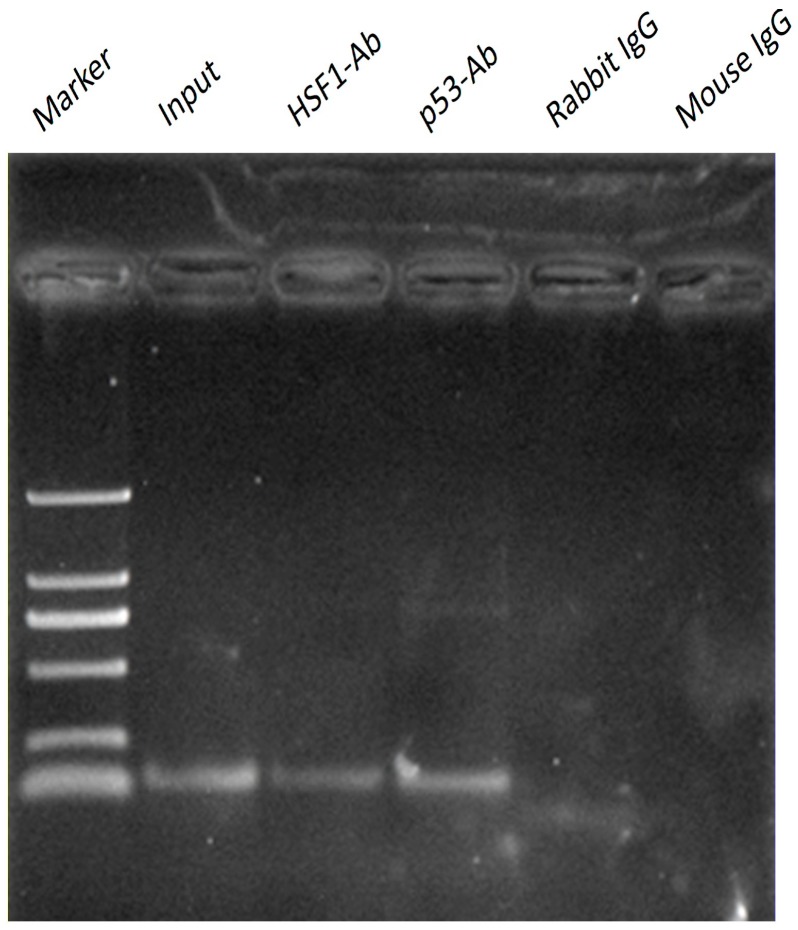
Chromatin Immunoprecipitation(ChIP) was performed with chromatin prepared from mouse heart. The promoter region containing the Heat shock transcription factor 1 (HSF1) and p53 site was analysed by PCR following immunoprecipitation with the HSF1 and p53 antibodies. Results of amplification for soluble chromatin before immunoprecipitation are shown as positive control (input) and negative control (IgG).

To examined whether p53 or HSF1 binds to the Omi/HtrA2 promoter, ChIP assays were carried out using mouse heart. We next reconfirmed the interaction of p53 or HSF1 with the Omi/HtrA2 promoter *in vivo* by ChIP assay. A p53 and HSF1 sense and antisense primer pair containing the p53 and HSF1 region showed a positive PCR signal in heart using anti-p53/HSF1 antibodies (Figure 4). This result suggests that p53 and HSF1 can bind to Omi/HtrA2 promoter as transcriptional factors.

## 3. Discussion

We have cloned and characterized the promoter of the mouse *Omi*/*HtrA2* gene, which encodes a serine protease. Previous studies reported that Omi/HtrA2 is a mitochondrial pro-apoptotic protein, Omi/HtrA2 promoted apoptosis though translocation from the mitochondria to the cytosol [14], regulated protein quality control, and maintained mitochondrial homeostasis [15,16]. The mutation Ser276Cys of Omi/HtrA2 and loss of Omi protease activity increased mitophagy in mnd2 mouse and Parkinson s disease(PD) in humans [17,18,19]. We previously reported that the release of Omi/HtrA2 from the mitochondria is a major factor in myocardial ischemia reperfusion injury [11]. Overexpression of Omi/HtrA2 contributes to apoptosis and increases the susceptibility of myocardial ischemia reperfusion injury in aging rat hearts [13]. Furthermore, Omi/HtrA2 expression and function is associated with autophagy and mitophagy in Parkinson’s [19,20] and Huntington’s disease [21] , and it is known to be upregulated in a variety of cancers [1,2,3,4,5,6,7,8,9,10]. To evaluate Omi/HtrA2 expression patterns, we performed Western blotting and QRT-PCR for a panel of different tissues. The expression and distribution of Omi/HtrA2 varied among different organs, with high expression in the brain, heart, liver and kidney, and low expression in the lung and spleen. Furthermore, the peak expression shifted from the kidney and liver to the heart and brain in aging mice. The observed patterns of expression were consistent at the level of protein and mRNA. These results suggest that the regulation of Omi/HtrA2 is complex and predominately occurs at the level of transcription.

Promoter sequences, located directly upstream of genes, are known to provide control points for the regulation of transcription. Promoters encompass multiple transcriptional binding sites, which can bind transcription factors and influence the expression level of mRNAs. To evaluate the mouse Omi/HtrA2 promoter, we amplified a 1298 bp containing the commercial product (MPRM17388, Genecopoeia, Rockville, MD, USA) and prepared several truncation mutations, which we ligated proximal to a luciferase reporter gene. The results demonstrated that −359 to 310 bp and 1197 to 1148 bp may be core regions of the Omi/HtrA2 promoter. Furthermore, the −709~+37 bp region is predicted to constitute a CpG island. The latter prediction presumes that DNA Methylation of the Omi/HtrA2 promoter is likely to be an important factor in regulating Omi/HtrA2 mRNA levels.

Notably, bioinformatic predictions for the Omi/HtrA2 active regions were consistent with the functional data obtained from luciferase assays. The RLA of pGL3-1298 (−1205~+93 bp) and pGL3-239 (−146~+93 bp) was significantly higher than pGL3-931 (−838~+93 bp) and pGL3-basic,, suggesting that −1205~−838 bp and −146~+93 bp comprise an active region of the Omi/HtrA2 promoter; consistently, −1197~−1148 bp was predicted as an active region by bioinformatics analysis. The activity of pGL3-931 (−838~+93 bp) was lower than pGL3-742 (−649~+93 bp), suggesting that −838~−649 bp region might contain an inhibitory *cis*-acting element or constitute a CpG island.

The bioinformatics analysis using TFSEARCH, PROMO and JAPAR identified multiple transcriptional binding sites on the Omi/HtrA2 promoter, including sites for HSF1, SP1, AP1, p53, and YY1. The identification of the HSF1 binding sites has particular potential importance for Omi/HtrA2 regulation. HSF1 is a heat shock protein transcription factor, which is related to cell differentiation, stress, aging and carcinogenesis. HSF1 is a master regulator of genes encoding molecular chaperones and is involved in cellular processes such as the stress response, cellular differentiation, aging and carcinogenesis. Suppression of HSF1 affects the advancement or maintenance of DNA damage signaling-induced cell senescence [20]. The inactivation of HSF1 leads to remarkable reduction in the transcript levels of its target genes, including heat shock protein family A (Hsp70) member 1A (HSPA1A (Hsp70)), DnaJ heat shock protein family (Hsp40) member B1(DNAJB1 (Hsp40)), and heat shock protein 90kDa alpha family class A member 1 (HSP90AA1) [21,22]. In *C. elegans*, HSF1 mediates chaperone activity in muscle cells and neurons and is associated with aging [23].

The other binding sites identified within the Omi/HtrA2 promoter also have potential functional significance. AP1 can induce myocardium apoptosis through the HO-1 pathway [24,25]. Decreased expression of AP1 inhibits apoptosis in the aging brain [26]_._ HSF1 regulates jun proto-oncogene(JUN) expression by binding to the JUN promoter, thereby modulating the activity of the transcriptional activator protein 1 (AP1) [27]. Therefore, the occurrence of both HSF1 and AP1 sites within the Omi/HtrA2 promoter could serve to amplify the regulatory effect. p53 modulates neuronal apoptosis, partially by suppressing the anti-apoptotic protein X-linked inhibitor of apoptosis protein (XIAP) via transcriptional activation of Omi/HtrA2 [28]. p53 induces activation of mitochondrial Omi/HtrA2 and blocks Ras-driven invasion by regulating the actin cytoskeleton [29]. YY1, histone deacetylase 3 (HDAC3) and histone deacetylase 4 (HDAC4) inhibit cell senescence by restraining p16 (INK4a) expression through epigenetic modification of histones [30]. The combination of these different binding sites within the Omi/HtrA2 promoter likely explains the complex pattern of tissue-specific expression.

HSF1 and p53 contain multiple predicted binding sites within the Omi/HtrA2 promoter, especially in its core regions at −1205~−838 bp and −146~+93 bp, which may be the reason for the activity of these regions. Furthermore, our study confirmed that HSF1 and p53 can bind to Omi/HtrA2 promoter by ChIP assay, and are likely to be important transcriptional factors involved in regulating Omi/HtrA2 mRNA levels.

## 4. Materials and Methods

### 4.1. Animals

C57BL/6 male mice were purchased from Suzhou Ai Er Mai Te Technology Co., LTD (license No.: SCXK [Su] 2014-0007) (Suzhou, China) and used at 6 months of age (“young”) or 24 months of age (“aging”). All the animal experiments followed the *Guiding Principles for the Use and Care of Animals* published by the National Institutes of Health (NIH publication no. 85-23, revised 1996).

### 4.2. Cell Lines and Cell Culture

NIH3T3, H9c2, and HEK293 cell lines were purchased from the Basic Medical Cell Center of Peking Union Medical College (Beijing, China). H9c2 and HEK-293 cells were cultured in Dulbecco’s modified Eagle’s medium (high glucose) with 10% fetal calf serum. NIH3T3 cells were grown in Dulbecco’s modified Eagle’s medium (high glucose) with 10% calf serum. The cells were cultured at 37 °C and 5% CO_2_.

### 4.3. Isolation of Genomic DNA

Genomic DNA was extracted from adult mouse hearts using the Universal Genomic DNA Extraction Kit Ver. 3.0 (#DV811A; Takara, Dalian, China). The concentration and purity of DNA was assessed using an ultramicro spectrophotometer (Implen GmbH, München, Germany). DNA samples with OD_260_/OD_280_ ratios of 1.8–2.0 were selected for subsequent experiments.

### 4.4. Design and Preparation of Mouse Omi/HtrA2 Gene Promoter Constructs

To prepare a full-length Omi/HtrA2 promoter luciferase constructs, we amplified a 1298 bp fragment (−1205 to +93 bp), which includes the mouse *Omi*/*HtrA2* gene promoter sequence, together with the start site of the NCBI gene (MPRM17388, Genecopia, Rockville, MD, USA,). To prepare promoter deletions, DNA fragments of different sizes were amplified using a shared reverse primer (R) and 5 different forward PCR primers: F1, 239 bp (−146~+93 bp), F2, 472 bp (−379~+93 bp), F3, 742 bp (−649~+93 bp), F4, 931 bp (−838~+93 bp), and F5, 1298 bp (−1205 ~+93 bp). The primer sequences are shown in Table 1.

The DNA fragments were amplified using PrimeSTAR^®^ HS DNA Polymerase (#DR010A; Takara) in a 50 µL reaction mixture containing 10 μL 5× PrimeSTAR Buffer (Mg^2+^ plus), 1 μL PrimeSTAR HS DNA Polymerase (2.5 U/μL), 4 μL dNTP Mixture (2.5 mM each), 1 µL of each primer (10 µmol/L), and 1 µL of DNA template (<1 µg). The PCR amplification protocol was as follows: denaturation at 94 °C for 2 min; 35 cycles of denaturation at 95 °C for 30 s, annealing at 62.4 °C for 45 s, and extension at 68 °C for 30–90 s (according to the fragment length of PCR products, about 1 kb/1 min); and final extension at 68 °C for 10 min. Samples were stored at 4 °C. All PCR products were verified by agarose gel electrophoresis and sequencing.

PCR products were purified using a DNA gel extraction kit (#AP-GX-50; Axygen, Corning, NY, USA), and then sticky end products were produced using a DNA A-Tailing Kit (Tiangen, Beijing, China) with a 20 μL reaction mixture containing 15 μL purified PCR products, 4 μL A-Tailing Mix, and 1 μL A-Tailing Enzyme (2.5 U/μL). The reaction mixture was incubated at 72 °C for 30 min and then stored at 4 °C. The A-tailing products were cloned into pMD18-T vector (#6011; Takara) to produce recombinant constructs (pMD18-T-239 to pMD18-T-1298) which were confirmed by sequencing.

Recombinant constructs pMD18-T-239 to pMD18-T-1298 and pGL3 luciferase reporter vector (#E1751; Promega, Madison, WI, USA) were digested for 4h at 37 °C with *Kpn*I (#1068A; Takara) and *Hind*III (#1060A; Takara), and the fragments of the recombinant constructs were subcloned into pGL3. The ligation mixtures, including 0.3 μg of recombinant fragments (*Kpn*I/*Hind*III), 0.1 μg of pGL3-basic plasmid (*Kpn*I/*Hind*III), 1 μL of 10× buffer M, and 1U of T4 ligase in a final volume of 10 μL, were incubated at 16 °C overnight and then 65 °C for 5 min. The ligation mixtures were transformed into *E. coli* DH5α cells. The positive recombinant clones (pGL3-239, pGL3-472, pGL3-742, pGL3-931, and pGL3-1298; designated on the basis of their target fragment lengths) were extracted with a plasmid mini kit (#AP-MN-P-50; Axygen) according to the manufacturer’s instructions. Recombinant plasmids were verified by sequencing and double digestion with *Kpn*I and *Hind*III.

### 4.5. Transfection and Luciferase Activity Assay

Cells were inoculated in 96-well plates at a density of 5 × 10^4^ cells/well and randomly divided into 7 groups: control (no transfection), pGL3-basic (no insert), pGL3-239, pGL3-472, pGL3-742, pGL3-931, and pGL3-1298. The medium was aspirated and replaced with serum-free medium without antibiotics 4 h before transfection according to the protocol of Lipofectamine 2000 (#11668-027; Invitrogen, Carlsbad, CA, USA). Lipofectamine 2000 (0.4 µL) was diluted with 25 µL Opti-MEM; and 100 ng of plasmid encoding Firefly luciferase and 10 ng of internal control plasmid encoding Renilla luciferase pRL-TK (#E2241; Promega) were diluted with 25 µL Opti-MEM^®^ Reduced-Serum Medium (#31985; Gibco, Carlsbad, CA, USA). The mixtures were incubated for 20 min at room temperature and then were added to the cells with antibiotic-free complete medium. Cell lysates were harvested 48 h after transfection. Firefly luciferase and Renilla luciferase activity were tested using the Dual-Glo^®^ Luciferase Assay System (#E2920; Promega) according to the manufacturer’s instructions. Firefly and Renilla luciferase activity were measured using a GloMax^®^ 96 Microplate Luminometer (Promega, Madison, WI, USA), and the relative luciferase activity (RLA) was determined by calculating their ratio.

### 4.6. Bioinformatics Analysis

In order to estimate the characteristic of the mouse Omi/HtrA2 promoter, bioinformatics analysis was done using online tools. The core activity of the mouse Omi/HtrA2 promoter was predicted by Network Promoter Prediction (Avalible online: http://www.fruitfly.org/seq_tools/promoter.html). The CpG island of the mouse Omi/HtrA2 promoter was predicted by CpG Island Searcher (Avalible online: http://cpgislands.usc.edu/) with lower limit values of the Observed/Expected ratio > 0.60 and GC content >50%. Transcription factors and their binding sites in the Omi/HtrA2 promoter were predicted by TFSEARCH (Avalible online: http://www.cbrc.jp/research/db/TFSEARCH.html), JAPAR (Avalible online: http://jaspar.genereg.net/cgi-bin/jaspar_db.pl?rm=browse&db=core&tax_group=vertebrates) and PROMO (Avalible online: http://alggen.lsi.upc.es/cgi-bin/promo_v3/promo/promoinit.cgi?dirDB=TF_8.3).

### 4.7. Western Blotting 

Protein lysates from tissues were prepared as previously described [13], and protein concentrations of lysates were determined using a BCA Protein Assay Kit (#23225; Thermo Scientific Pierce, Carlsbad, CA, USA). Fifty µg/lane of each sample were run on 10% sodium dodecyl sulfatepolyacrylamide gel electrophoresis (SDS-PAGE) gels, depending of the target proteins, and then were electrotransferred onto polyvinylidene fluoride membranes. The membranes were incubated with primary antibodies anti-Omi/HtrA2 (#2176; 1:1000; Cell Signaling Technology, Danvers, MA, USA) or rabbit anti-glyceraldehyde-3-phosphate dehydrogenase/GAPDH (#2118; 1:1000; Cell Signaling Technology, Danvers, MA, USA) overnight at 4 °C, followed by incubation with the matched secondary antibodies for 1h at room temperature. The density of the target protein bands was measured using ImageJ software, and GAPDH was used as a protein loading control.

### 4.8. RNA Preparation and Quantitative Reverse Transcription-Polymerase Chain Reaction (QRT-PCR) Analysis

Total RNA was extracted from different tissues (heart, brain, liver, kidney, lungs, and spleen) in adult and aging mouse hearts using the RNAprep pure Tissue Kit (#DP431; Tiangen). The first cDNA strand was synthesized using a 1st-Strand cDNA Synthesis Kit (RR036A; Takara), and then real time PCR was performed using the SYBR PrimeScript RT-PCR Kit (RR820A; Takara). All of the procedures were performed according to the manufacturer’s instructions. Primers of mouse Omi/HtrA2 and GAPDH for QRT-PCR (SYBR) were designed and synthesized as follow: GAPDH-F: 5’-CTCTCTGCTCCTCCCTGTTCC-3’; GAPDH-R, 5’-CGTTCACACCGACCTTCACC-3’; Omi-F, 5’-ATCTCCTTTGCCATCCCTTC-3’; Omi-R, 5’-GGTCAGCATCATCACTCCAA-3’. The expression of Omi/HtrA2 mRNA in different tissues was calculated by the 2^−ΔΔ*C*t^ method, with GAPDH as internal control.

### 4.9. ChIP Assay

The ChIP assay was performed according to the instructions of the Magna ChIP G Tissue Kit (#17-20000; millipore EZ-ChIP, Darmstadt, Germany). Briefly, mouse heart were treated with 1% (*w*/*v*) formaldehyde for 15 min. Cross-linked chromatin was then prepared and sonicated to an average size of 500 bp. DNA fragments were immunoprecipitated with antibodies specific to HSF1 (#4356; Cell Signaling Technology, Danvers, MA, USA), p53 (#ab28; Abcam, Cambridge, UK), or control rabbit, mouse IgG at 4 °C overnight. After reversal of cross-linking, the immunoprecipitated chromatin was amplified by primers corresponding to core regions of the Omi/HtrA2 promoter. Primers were designed and synthesized as follow: Omi promoter-F, 5’-GCTACCGTCGTGCCCTGCTT-3’; Omi promoter-R, 5’-ATGCCCGAAGGCTCCAGTTT-3’.

### 4.10. Statistical Analysis

All values in the text and figures are expressed as mean ± SD. Significant differences between two transfection group were subjected to ANOVA and followed by post-hoc Dunn’s multiple comparison test. Western blot densities and QRT-PCR were analyzed using the Kruskal–Wallis test followed by Dunn’s *post hoc* test.

## 5. Conclusions

The tissue-specific expression of Omi/HtrA2 and its changing trend in aging mice was demonstrated in our study. The Omi/HtrA2 promoter’s active region was mapped from −1205 to −838 bp and −146 to +93 bp, with the −838 to −649 bp region exhibiting negative regulatory activity according to bioinformatics analysis and a dual-luciferase reporter assay system. A CpG island was predicted at −709 to +37 bp of the Omi/HtrA2 promoter. On the basis of these findings, the Omi/HtrA2 promoter may be methylated and include transcriptional binding sites (HSF1, SP1, AP1, p53, and YY1). HSF1 and p53 can bind to Omi/HtrA2 promoter, and are likely to be important transcriptional factors in regulating Omi/HtrA2 mRNA levels.

## Supplementary Materials

Supplementary materials can be found at http://www.mdpi.com/1422-0067/17/1/119/s1.
