# Supplementary material for: Cloning and Transcriptional Activity of the Mouse Omi/HtrA2 Gene Promoter
Source: Int J Mol Sci. 2016 Jan 16;17(1):119. doi: 10.3390/ijms17010119 (PMC4730360; doi:10.3390/ijms17010119)
Supplement: Supplementary file 1 [file ijms-17-00119-s001.pdf]

Supplementary Materials: Cloning and Transcriptional Activity of the Mouse *Omi*/*HtrA2* Gene Promoter

Dan Liu, Xin Liu, Ye Wu, Wen Wang, Xinliang Ma and Huirong Liu

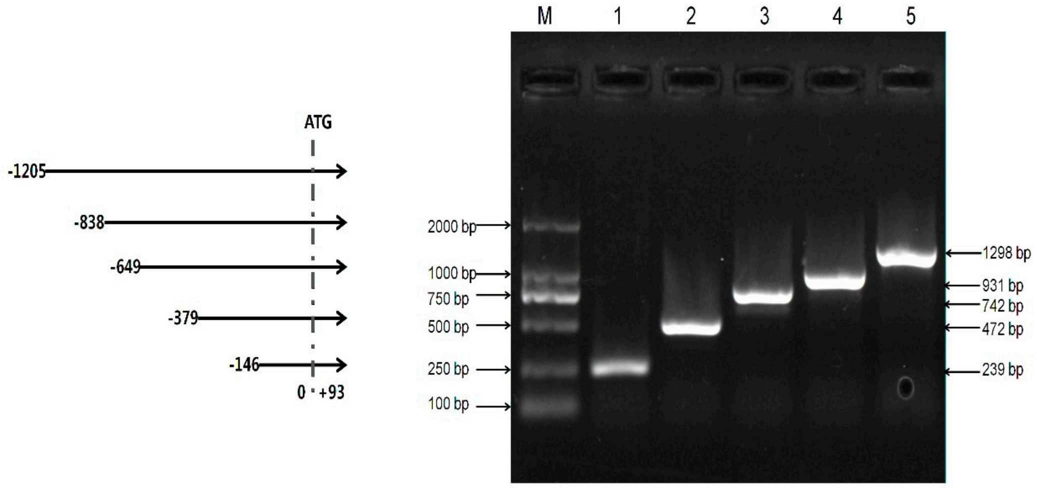

Figure S1. PCR amplification of the mouse *Omi*/*HtrA2* gene promoter. *Omi*/*HtrA2* promoter fragments were amplified using F1–F5 as upstream primers and R as a downstream primer. M: DL2000 Marker; **1**: 239 bp; **2**: 472 bp; **3**: 742 bp; **4**: 931 bp; **5**: 1298 bp.
